# Northern European *Salmo trutta* (L.) populations are genetically divergent across geographical regions and environmental gradients

**DOI:** 10.1111/eva.12877

**Published:** 2019-11-01

**Authors:** Dorte Bekkevold, Johan Höjesjö, Einar Eg Nielsen, David Aldvén, Thomas Damm Als, Marte Sodeland, Matthew Peter Kent, Sigbjørn Lien, Michael Møller Hansen

**Affiliations:** ^1^ National Institute of Aquatic Resources Technical University of Denmark Silkeborg Denmark; ^2^ Department of Biological & Environmental Sciences University of Gothenburg Gothenburg Sweden; ^3^ Generations R&D Vattenfall AB Älvkarleby Sweden; ^4^ Department of Biomedicine Aarhus University Aarhus Denmark; ^5^ Department of Natural Sciences University of Agder Kristiansand Norway; ^6^ Faculty of Biosciences Norwegian University of Life Sciences Ås Norway; ^7^ Department of Bioscience – Genetics, Ecology and Evolution Aarhus University Aarhus Denmark

**Keywords:** brown trout, genotype‐environment association, local adaptation, outlier test, salmonid

## Abstract

The salmonid fish Brown trout is iconic as a model for the application of conservation genetics to understand and manage local interspecific variation. However, there is still scant information about relationships between local and large‐scale population structure, and to what extent geographical and environmental variables are associated with barriers to gene flow. We used information from 3,782 mapped SNPs developed for the present study and conducted outlier tests and gene–environment association (GEA) analyses in order to examine drivers of population structure. Analyses comprised >2,600 fish from 72 riverine populations spanning a central part of the species' distribution in northern Europe. We report hitherto unidentified genetic breaks in population structure, indicating strong barriers to gene flow. GEA loci were widely spread across genomic regions and showed correlations with climatic, abiotic and geographical parameters. In some cases, individual loci showed consistent GEA across the geographical regions Britain, Europe and Scandinavia. In other cases, correlations were observed only within a sub‐set of regions, suggesting that locus‐specific variation was associated with local processes. A paired‐population sampling design allowed us to evaluate sampling effects on detection of outlier loci and GEA. Two widely applied methods for outlier detection (*pcadapt* and *bayescan*) showed low overlap in loci identified as statistical outliers across sub‐sets of data. Two GEA analytical approaches (LFMM and RDA) showed good correspondence concerning loci associated with specific variables, but LFMM identified five times more statistically significant associations than RDA. Our results emphasize the importance of carefully considering the statistical methods applied for the hypotheses being tested in outlier analysis. Sampling design may have lower impact on results if the objective is to identify GEA loci and their population distribution. Our study provides new insights into trout populations, and results have direct management implications in serving as a tool for identification of conservation units.

## 1 INTRODUCTION

Improving our understanding of the genetic basis of local adaptation is a main aim in evolutionary biology and is also of significance in applied research because of its relevance to the conservation of genetic resources, management of exploited populations and for predicting impacts of climate change (Allendorf, Hohenlohe, & Luikart, 2010; Lehnert et al., 2019). Traits that confer local adaptation are typically polygenic quantitative traits, and identification of the loci that determine variation in such traits is usually challenging (Savolainen, Lascoux, & Merilä, 2015). Testing hypotheses of local selective sweeps and their association with environmental drivers by means of scanning genomic profiles across diverged populations provides novel insights but has also been criticized, as a range of factors may obscure or lead to false‐positive inference about adaptive processes and the ecological mechanisms that structure populations (Ahrens et al., 2018). Demographic processes may for instance obscure inference about selection and the role of the environment in driving spatial patterns of adaptation (de Villemereuil, Frichot, Bazin, Francois, & Gaggiotti, 2014). There is a general call for evaluation of statistical methods (Vatsiou, Bazin, & Gaggiotti, 2016), particularly for populations connected by gene flow (Bradburd, Coop, & Ralph, 2018; Luu, Bazin, & Blum, 2017). It has been suggested that in analyses of gene–environment associations (GEA), sampling multiple populations exposed to similar environmental conditions is a means to increase detection power of true positives, especially for associations with weakly selected loci (Lotterhos & Whitlock, 2015). However, studies applying such sampling design are still rare (Roschanski et al., 2016) and tend to be restricted to local geographical scales (Ahrens et al., 2018).

Brown trout, *Salmo trutta* (in its anadromous form known as sea trout), is an ecologically and socioeconomically important salmonid fish species that allows for testing sampling effects on detection of local selective sweeps. Owing to its extreme ecological adaptability, it shows a widespread distribution throughout freshwater systems in most north‐east Atlantic and western Asian regions (Klemetsen et al., 2003). The species is considered as an indicator of habitat quality in its native range (Imhof, Fitzgibbon, & Annable, 1994), and there are concerns about its conservation status under a range of anthropogenic stressors and climate change (Ayllón et al., 2016). Since the first paper by Allendorf, Ryman, Stennek, and Stahl (1976), four decades of genetic marker‐based studies have identified genetically differentiated local populations of brown trout, sometimes even at small (<1 km) spatial scales (Ferguson, 1989; see Andersson et al., 2017 for a recent example). Although sea trout is renowned for natal homing, straying between rivers maintains gene flow and reduces the impact of genetic drift on population demographics (Hansen, 2002; Hansen, Fraser, Meier, & Mensberg, 2009). A suite of studies has suggested that local adaptation plays a role in population structuring and dynamics (see Meier, Hansen, Bekkevold, Skaala, & Mensberg, 2011 and references herein). As a poikilotherm, temperature directly affects the rate of biological processes and trout is expected to display evolutionary adaptations to reach homoeostasis. Coupled with an anadromous life cycle, which for the iteroparous brown trout may entail several repeated movements between fresh, brackish and marine waters, trout is required to both adapt to local conditions while still retaining the capability for coping with strongly varying environments. Altogether, these characteristics render the species an optimal model for testing ecological and evolutionary parameters, including local adaptation and its association with specific environments (Jensen et al., 2008).

In spite of brown trout being among the best studied fish species (Klemetsen et al., 2003), genomic resources have until recently been scarce compared to other salmonids, such as Atlantic salmon (*S. salar* L.) and rainbow trout (*Oncorhynchus mykiss* W.), which has hampered the study of GEA. Genomic resources for brown trout are developing rapidly. Nonetheless, genome‐wide SNP analyses have hitherto not been applied to examine broadscale population genetic relationships and associations between genomic variation and evolutionary drivers across spatial scales. Thus, to date there has been no assessment of genome‐wide population structure beyond geographically restricted populations, limiting our understanding of the processes determining large‐scale population connectivity. Evidence for local adaptation is commonly based on comparisons of populations at local scales (Andersson et al., 2017; Lemopoulos, Uusi‐Heikkilä, Huusko, Vasemägi, & Vainikki, 2018; Meier et al., 2011), while assessments rarely concomitantly address small‐ and large‐scale patterns. Lack of knowledge about ecological drivers of population processes is especially problematic given that many trout populations are considered under threat due to disturbances acting on both large scale, for example climate change (Jacquin et al., 2017; Pujolar, Vincenzi, Zane, & Crivelli, 2016; Vera, Martinez, & Bouza, 2018), and local scale. For example, anthropogenic habitat destruction, creation of impassable dams preventing gene flow (Hansen, Limborg, Ferchaud, & Pujolar, 2014) and genetic introgression from widespread stocking with non‐native strains (Gil, Labonne, & Caudron, 2016; Hansen et al., 2009) is expected to affect a wide number of populations.

In order to investigate population differentiation and its potential environmental drivers in brown trout using genome‐wide analysis, we here developed and applied a SNP array encompassing ca. 3.8K mapped SNPs. In GEA analyses, populations showing hierarchically structured levels of gene flow (as in brown trout) can lead to obscured or false‐positive inference about adaptive processes and the ecological drivers of diversification (Ahrens et al., 2018; Bradburd et al., 2018; Forester, Lasky, Wagner, & Urban, 2018; Luu et al., 2017). Following Lotterhos and Whitlock (2015), we therefore applied a paired‐population sampling approach in order to analyse 72 *S. trutta* populations, spanning a central part of the distribution of anadromous populations in Europe in order to first, describe regional scale population structure, and second, determine whether population divergence was associated with GEA and selective sweeps. Concomitantly, our study was designed to evaluate effects of sampling design and analytical approach on detection of outlier loci and GEA in a hierarchical population scenario. The aim was to contribute new insights on environmental selection pressures in general and in anadromous fish species in particular, and at the same time add to the knowledge of population structure of *S. trutta*, a key indicator species for the health and conservation of rivers and streams.

## 2 MATERIALS AND METHODS

### 2.1 Population samples

A SNP development panel was built using genomic DNA from 2 to 3 fish from each of seven ascertainment populations (Table 1). Populations were spread out geographically so as to span the region from the British Isles in west to the Baltic Sea in east (>1,500 km), and from Norway in north to the Wadden Sea in the southern North Sea (>1,000 km). All ascertainment samples represented the species' Atlantic clade (Bernatchez, 2001) and covered the geographical area represented by population collections in the present study.

**Table 1:** Collections of *Salmo trutta* indicating country, geographical region and river, where number refers to map location in Figure 1

Country	Region (Sea)	Rivers (number in Figure 1)
Norway	W Scandinavian peninsula (Hardangerfjord, North Sea)	1. Granvin^a^, 2. Guddal
United Kingdom	Britain, northeast (North Sea)	3. Spey, 4. Deveron, 5. Eyewater, 6. Tweed, 7. Aln, 8. Coquet, 9. Tyne, 10. Wear, 11. Tees, 12. Esk, 13. Ure
	Britain, southeast (North Sea)	14. Stiffkey, 15. Glaven, 16. Nar
	Britain, Cornwall (English Channel)	Tamar^a^
Germany	Continental Europe, Jutland Peninsula, Wadden Sea (North Sea)	17. Weser, 18. Elbe
Denmark		19. Ribe^a^, ^b^, 20. Kongeå^b^, 21. Sneum^b^, 22. Varde b
	Continental Europe, Jutland Peninsula (North Sea)	23. Skjern b, 24. Storå^b^, 25. Liver^b^
	Continental Europe, Jutland Peninsula (Limfjord, Kattegat)	26. Simested, 27. Jordbro, 28. Skals, 29. Karup^a^
Norway	E Scandinavian Peninsula (Skagerrak)	30. Sonsbeck
Sweden		31. Krokstrand, 32. Hogar/Strommeå, 33. Anråsälv, 34. Bärfendalsbäcken, 35. Broälv, 36. Taskeå, 37. Karraå, 38. Bodelå, 39. Henån, 40. Bratteforsan, 41. Norumsån, 42. Säbyån, 43. Grannebyån, 44. Sörån
	E Scandinavian Peninsula (Kattegat)	45. Krogarebäcken, 46. Hallebäcken, 47. Himleån, 48. Ätran & Högvadsån, 49. Fylleån
Denmark	Continental Europe, Jutland Peninsula (Kattegat)	50. Elling, 51. Villestrup å, 52. Lilleå a, 53. Hevring, 54. Grenå, 55. Lake Hald, 56. Lake Mossø, 57. Giber å^b^
	Continental Europe, Jutland Peninsula (W Belt Sea)	58. Kolding, 59. Tapså, 60. Adsbøl
	Continental Europe, Belt Sea Islands (Belt Sea)	61. Stokkebæk^b^, 62. Saltø b, 63. Fladså^b^, 64. Mern, 65. Krobæk
	Continental Europe, Bornholm Island (W Baltic Sea)	66. Tejn a, 67. Læså
Estonia	Continental Europe, Gulf of Finland (E Baltic Sea)	Vainupea
France	Haute‐Savoie, Continental Europe, Mediterranean Sea	Rhone tributary Les Usses
Denmark	Domesticated hatchery strains	HAT1, HAT2

Note

Rivers with temporal replicates are underlined. Detailed sample information is given in Table S1.

a Samples included in the SNP development ascertainment panel.

b Populations exhibiting introgression from one or both partially domesticated hatchery strains.

**Figure 1:**
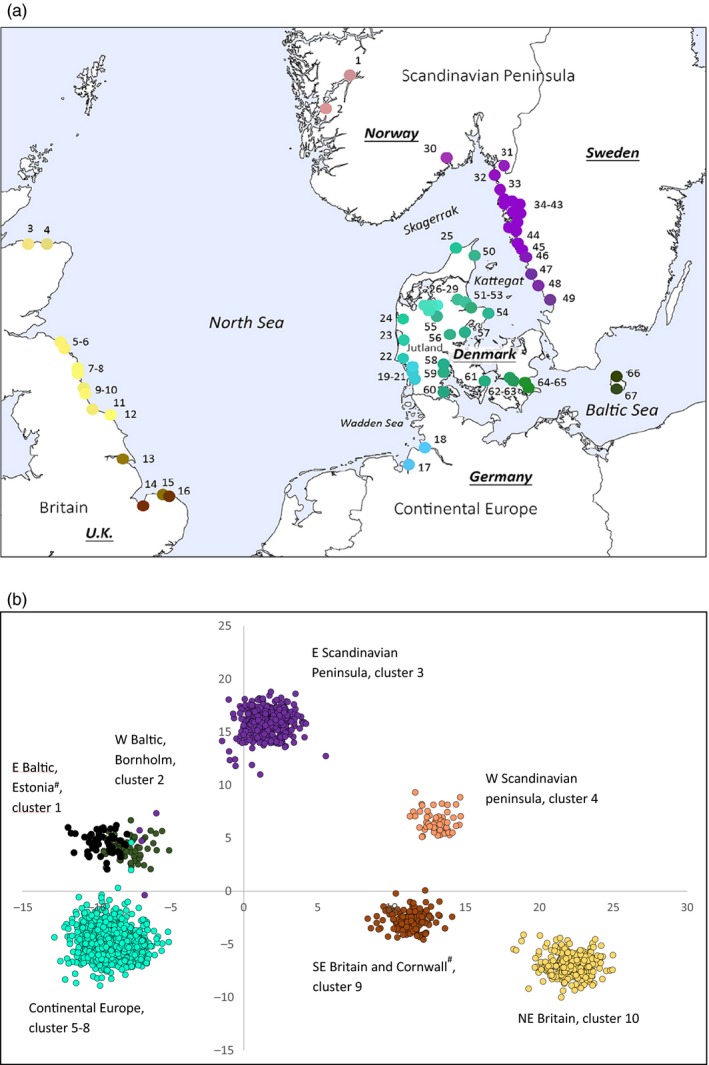
(a) *Salmo trutta* populations in the analysis (extant collections from France, Estonia and Cornwall are not shown on map) indicated by individual rivers' confluence with the sea, except for lake trout samples 55 and 56. Numbers refer to Table 1. Symbol colours reflect genetic clustering, where RGB colour code is determined by the first three axes in the principal component analysis. (b) Discriminant analysis of principal components clustering of individual genotypes at *k* = 10 for Discriminant functions 1 and 2, describing, respectively, 37% and 20% of the variance. Geographical areas corresponding to genotype clusters are indicated by symbol colour and text. Geographically distant collections are indicated by #. Estonia forms a separate cluster (black), whereas the collection from Cornwall clustered with SE British samples

To describe population genomic patterns, trout were collected from 74 spawning locations in 72 rivers (on average 36 fish collected per river; Table 1) draining into the North Sea, Skagerrak, Kattegat and the Western Baltic Sea (Figure 1). In analyses of GEA, sampling multiple populations exposed to similar environmental conditions represents a means to increase detection power of true positives, especially for associations including weakly selected loci (Lotterhos & Whitlock, 2015). We therefore aimed to sample a minimum of two rivers from each geographical sub‐area. Sub‐areas were here defined as geographically proximate river systems expected to share environmental drivers. When individual rivers show some degree of demographic isolation, as is the assumption in a natal‐homing anadromous fish, the paired‐sampling approach thus represents a way to cross‐validate identified GEA loci. Sampling multiple rivers per sub‐area was not possible in all cases. Data therefore included four collections each representing only a single sub‐region. Eight locations were sampled twice, 5–14 years apart, to examine temporal stability. Collections consisted of adipose fin clips from electrofished, anaesthetized adults caught on spawning sites, or of 0–1 year juveniles, depending on availability (Table 1; Table S1). The River Ätran was represented by two collections, one from the main stem and one from its tributary Högvadsån. For the River Weser, collections of 5–9 fish from each of three neighbouring tributaries were combined. Sampling was aimed at natural populations with gene pools presumed to be relatively weakly affected by human‐mediated introgression from farmed or foreign populations. However, several of the Danish populations were previously stocked with two closely related hatchery strains, leading to admixture and introgression (Hansen et al., 2009). Samples from the stocked hatchery strains were therefore also included in analyses in order to identify potential impact of introgression (Table 1).

To compare regional structuring with patterns at broader geographical scales, collections also included three geographically remote populations: the Estonian Vainupea River (draining into the Gulf of Finland in the eastern Baltic Sea), River Tamar in Cornwall, UK (draining into the western English Channel), and from les Usses River, draining into the River Rhone in the Haute‐Savoie in southern France. The latter representing the species' Mediterranean clade (Bernatchez, 2001). DNA from all samples was extracted from adipose fin clips using a commercial kit (E.Z.N.A.™ kit; Omega BioTek).

### 2.2 SNP array development, genotyping and linkage analysis

A custom Illumina iSelect SNP array (with 6,000 SNPs) was developed by aligning reads in 16 male and female ascertainment samples against a draft genome assembly. Nextera sequencing libraries were prepared from genomic DNA and sequenced using a Illumina HiSeq 2000 to generate paired‐end reads (2 × 100 bp); between 5 and 13 Gb of sequence was generated for each individual (average 6.9 Gb, see Table S2). After filtering to remove adapter sequence, low‐quality sequence and any reads less than 60 bp, SOAPdenovo (Li et al., 2010) was used to generate a de novo assembly. Ignoring contigs <150 bp, the resulting fragmented assembly included 1,131,000 contigs (N50 = 2,281) and contained 1.4 Gb of sequence. The assembly was repeat masked using RepeatMasker software (http://www.repeatmasker.org/) and a locally developed salmonid‐specific repeat library developed with the RepeatModeler software. Reads from the 16 individuals were aligned to the reference using BWA (Li & Durbin, 2009); putative SNPs within each individual were identified using SAMtools (Li et al., 2009). Selection criteria required that heterozygous SNPs should be supported by minimum of two reads in two individuals for the minor allele, across all samples at least one individual should be homozygous with a minimum of four reads, and only bi‐allelic SNPs separated by >60bp to the nearest indel or SNP were considered. A list of 47,380 putative SNPs was initially reduced by considering factors such as read depth, proximity to repeats and Illumina's Assay Design Score. Subsequent filtering prioritized SNPs based on whether they mapped to full‐length cDNA sequences/contigs from Atlantic salmon (Lien et al., 2011) were distributed across larger (>7,750 bp) brown trout contigs or fell within specific genes of interest.

To order SNPs by linkage group (LG) and generate a linkage map, we used the 6K SNP array to genotype both the 2,606 population samples and family material consisting of 320 individuals applying the manufacturers' recommended protocol and by calling genotypes using GenomeStudio V2 (Illumina). Family material came from a study by Jensen et al. (2008) and consisted of 10 F1 offspring from each of 30 full‐ and half‐sib families based on a total of 10 females and 10 males from two Danish populations also sampled separately for the current study (Lilleå and Karup). A modified version of the CRIMAP 2.4 software (Green, Falls, & Crooks, 1990), including added utilities provided by Xuelu Liu and Michael Grosz (Monsanto, St. Louis, MO, USA), was used for the map construction. Initially, SNPs were assigned to LG based on pairwise linkages and the grouping algorithm implemented in the AUTOGROUP option of the programme. The analysis assigned 3,894 SNPs to 40 LGs which correspond to the expected karyotype of brown trout (Martínez et al., 1991). After their initial grouping, SNPs were ordered within LGs using the *BUILD* and *FLIPSN* options in CRIMAP. The *CHROMPIC* option in CRIMAP was then used to phase genotypes within LGs, and a custom‐made script was used to correct or remove erroneous genotypes based on unlikely tight double recombination events. Finally, multipoint linkage maps for the 40 LGs were constructed using the *FIXED* option of CRIMAP. SNPs that could be mapped to LG and that showed Mendelian segregation of genotypes in pedigree material were selected (excluding SNPs statistically deviating from expected proportions in chi‐square tests across >10% individuals). This resulted in the retention of information for 3,782 SNPs.

### 2.3 Population analyses

Global and per SNP observed and expected heterozygosity (*H_O_* and *H_E_*) were determined for each of the 84 collections (samples from 74 rivers, two hatchery strains and eight temporal replicates) using the R package *adegenet* (Jombart, 2008). Samples comprising siblings may bias allele frequency estimates (Hansen & Jensen, 2005). To avoid this, we used the maximum‐likelihood‐based method described in Wang (2013) to analyse genetic relationships in samples exhibiting statistically significant *F*_IS_ estimates. Individuals exhibiting relatedness levels corresponding with half‐ and full‐sib relationships were recorded, and data were subsequently trimmed to exclude any such related individuals. This meant that 19 of 40, mostly juvenile, fish from the River Ure were excluded from further analysis. Using adegenet, locus‐specific *F*_ST_ values were calculated across all samples and deviation from HWE within samples was tested using chi‐square testing. Linkage disequilibrium was estimated using the squared correlation between alleles at two loci, both per sample and averaging across samples, using the R package *genetics* (Warnes, 2013). *p*‐values of HWE tests were adjusted for multiple comparisons using false discovery rate (FDR; Benjamini & Hochberg, 1995). All loci were retained in initial analyses, but in GEA and outlier analyses only one SNP was retained when pairs of SNPs showed very high LD (*r^2^* > .8) across samples.

Estimates of population structure and detection of outlier loci may be downwardly biased if markers are selected using specific criteria about the numbers of times they occur in the SNP development sample (Rosenblum & Novembre, 2007). Here, ascertainment bias (AB) was potentially non‐negligible due to the relatively strict criteria applied when selecting candidates for the SNP array, particularly the criterion that at least two individuals from the ascertainment panel had to have the alternate allele in at least two reads. Potential effects of AB were assessed by inspection of minor allele frequencies per population sample, where the expected nonbiased distribution describes a beta distribution with high proportions of low‐frequency markers rapidly decreasing towards low proportions of high‐frequency markers.

Sample differentiation was described using pairwise ϴ (Weir & Cockerham, 1984). Isolation‐by‐distance (IBD) relationships were examined using a Mantel test and 999 randomizations in *adegenet*, including information for least waterway distance to river mouth in all spatial samples for all loci. Including all loci, clustering of samples was visualized using principal component analysis (PCA) and discriminant analysis of principal components (DAPC), following Jombart and Ahmed (2011). The French sample was excluded from these and subsequent analyses due to its highly divergent (Mediterranean lineage) origin. Bayesian information criterion (BIC) was used for evaluation of the partitioning of individuals into different number of clusters (*k*) in DAPC. Based on discriminant functions, individual posterior membership probabilities were subsequently evaluated to assess the suitability of the approach in terms of capturing correspondence between capture locality and genetic clustering.

### 2.4 Genome scan

A genome scan approach was used to distinguish genome‐wide processes expected to mainly reflect demographic histories, from processes at individual loci potentially reflecting local processes, particularly selection. Genome scans may suffer from inflated numbers of false positives under hierarchical spatial structure coupled with isolation‐by‐distance dynamics (Excoffier, Hofer, & Foll, 2009). We therefore used a principal component analysis with a Mahalanobis distance‐based approach to identify outlier loci (Luu et al., 2017). This method, implemented in the R package *pcadapt,* allows for an examination of how different levels of population clustering affect outlier detection. It is reported to yield increased power compared to Bayesian models, especially when there is hierarchical population structure under divergence and range expansion scenarios (Luu et al., 2017). Initially, 50 principal components (PC) were used to assess the best supported genetic clustering among sampled individuals, where the optimal number (or range) of PC was determined using Cattell's graphical rule, following Luu et al. (2017). Outliers were then detected applying different levels of sample clustering and FDR to control error. Throughout, loci with global MAF <0.05 were excluded. To examine effects of sampling specific populations on outlier detection, analyses were finally repeated for sub‐sets of data: (a) including one half of the geographically paired collections selected at random (*N* = 34), (b) including the other half of paired samples (*N* = 34) and (c) combining these two sets of data (*N* = 68; Table 1). In all three latter cases, information was excluded for temporal replicates and for the four sub‐regions for which only a single collection was available, including extant populations Tamar, Vainupea and Haute‐Savoie (Table 1). *pcadapt* results were compared with results generated with the often used Bayesian *bayescan* method (Foll & Gaggiotti, 2008). This method is expected to exhibit low false‐positive rates, particularly when many population samples can be included (Narum & Hess, 2011), which was the case here. Settings followed recommendations in Foll and Gaggiotti (2008), excluding information for 66 SNPs exhibiting global MAF below 0.05. Following Foll and Gaggiotti (2008), we used Jeffrey's scale of evidence and defined potentially selected loci as markers having log_10_ (PO) above 1, but excluded loci with *Q*‐values above 0.01, to minimize FDR bias.

### 2.5 Genotype‐environment association

Signatures indicative of local adaptation to environmental variables were investigated using the univariate GEA method implemented in LFMM v. 1.5 (Frichot, Schoville, Bouchard, & François, 2013). The approach uses latent factor mixed models that take into account neutral population structure when testing associations between gene variation and candidate environmental variables. Environmental variables are entered into the model as fixed effects while population structure is modelled using latent factors. We used population clustering results from DAPC and *pcadapt* to guide the choice of *k* latent factors, varying *k* 9–14, and running 10 replicates per factor level, with Gibbs sampling algorithm with 20K iterations, discarding the first 5K iterations as burn‐in. In the LFMM model, a matrix term models the part of genetic variation that cannot be explained by the environmental variables. Selective responses in anadromous brown trout populations are expected to be mainly governed by regional, rather than by small‐scale environmental drivers (Hansen, 2002). Accordingly, we examined variables expected to reflect effects of different types of drivers on genetic variation. Firstly, to control for IBD dynamics and postglacial founder events, we entered sample latitude and longitude, and whether origin was either the British Isles, European continent or Scandinavian Peninsula (as a categorical variable). Secondly, potential relationships with abiotic conditions on spawning sites were examined by entering data on geochemistry (using soil type and soil pH), annual average water temperature, minimum winter temperature and maximum summer temperature, annual temperature range, average annual precipitation and precipitation in driest month at collection site. Climate data were obtained from https://esdac.jrc.ec.europa.eu/. Data on soil type (entered as categorical variable), and pH values were obtained from http://eurosoils.jrc.ec.europa.eu/. Finally, to test for relationships between genetic variation and abiotic environment first encountered during smolt sea‐migrating stage, information was included on average ambient water temperature and salinity 1 km from river confluence with the sea (from http://marine.copernicus.eu/). Thirdly, we entered altitude at collection site as a proxy for both upstream spawning run and smolt downstream migration barrier. Fourthly, to examine effects of genetic introgression from stocked hatchery strains, we entered categorical information about whether populations had previously been stocked with either of the two hatchery strains (pertains to Danish populations). Associations among variables were tested using the function PCAMIX in the R package *PCAmixdata* (Chavent, Kuentz‐Simonet, Labenne, & Saracco, 2014) allowing for PCA of mixed qualitative and quantitative data. Based on eigenvalues for 24 dimensions and using Cattell's graphical rule, the first five principal components from that analysis were used to reduce variables tested in the model. Following Frichot et al. (2013), *p*‐values < 10^–5^ obtained after applying a Bonferroni correction for a type I error at *α* = .01 and ~10^–4^ loci, *z*‐scores >4.7 were considered to show GEA.

Recent simulation analyses suggest that multivariate GEA methods, such as redundancy analysis (RDA), under some conditions may perform better than PCA‐based methods in identifying environmental predictors of genotype variation and in selective outliers (Capblancq, Luu, Blum, & Bazin, 2018; Forester et al., 2018). RDA is a multivariate ordination approach that combines PCs from allele frequency and multivariate environmental distance matrices to produce canonical axes predicting relationships between environments and particular loci. We therefore compared results from LFMM with an RDA analysis using the *vegan* package (Oksanen et al., 2015) following the procedure detailed in Forester et al. (2018). Missing data were imputed using the most common genotype observed within samples. As for the LFMM analysis, the five environmental PCs were tested against SNP data in 68 sample collections. The function *vif.cca* was used to ascertain lack of multi‐collinearity among variables, as expected from the use of composite environmental variables, which alleviated the need for variable reduction. There was no a priori control for population structure. Following Forester et al. (2018), we classified SNPs as showing statistically significant association with individual environmental parameters when they loaded with more than three standard deviations from the mean. We estimated correlations between these SNPs and their most strongly associated environmental variable following Forester et al. (2018).

## 3 RESULTS

### 3.1 SNP array performance

All SNP clusters were visually inspected using Genome Studio and all markers subjectively classified according to sample data point clustering precision and accuracy. A total of around 4,000 markers (72%) displayed tight grouping into three well separated genotype clusters, 1,106 (20%) appeared to be monomorphic in the examined samples, and the remainder displayed clustering patterns making SNP genotype calling unreliable.

The 40 brown trout LGs with 3,894 SNPs represent, on average, 95 SNPs per LG (range 13–173) and adds up to a male map of 1,316 cM and a female map of 2,494 cM (Table S2 for details on linkage map, assembly and annotation). Relative to the expected beta distribution, MAFs were skewed towards high values (Figure S1). Average MAF was 0.28, and 1.5% of loci had global MAF <0.05 (Table S3). In total, 153 pairs of loci showed evidence of linkage disequilibrium with genotype associations at *R^2^* > .8.

### 3.2 Genetic relationships within and among populations

The 3,782 SNPs contained within the linkage map and conforming to Mendelian segregation rules were typed across >95% of the 2,536 individuals in the 84 sample collections. *H_E_* varied 0.31–0.37 across collections (average = 0.35) and tended to be slightly lower in collections from Britain (average ± *SD* = 0.33 ± 0.01) than in continental and Scandinavian populations (average ± *SD* = 0.36 ± 0.01; *t* test = 9.259, *df* = 79, *p* < .001). No other trends in *H_E_* were observed among geographical regions. Excluding information from extant populations, global, locus‐specific Ɵ values ranged between 0.007 and 0.237, with a slightly lower median (0.061) than mean value (0.067) (Table S4). Evidence for departure from HWE (at *α* = .05) was found in 4,541 of 150,066 tests, whereof nine remained significant after correction for multiple testing, none of which were particular to specific collections or loci. Global differentiation among sample collections was estimated at 0.068. The geographically distant sample collections Tamar in west and Vainupea in east both showed relatively strong differentiation from their geographically closest collections (Tamar‐Stiffkey Ɵ = 0.10; Vainupea‐Tejn Ɵ = 0.11). As expected, the les Usses sample representing the Mediterranean lineage showed the strongest differentiation of all, varying between 0.36 and 0.46 in pairwise comparisons (Table S4). Relatively low differentiation was observed between les Usses and the Danish hatchery strain #1, corresponding with the fact that this strain has been exported to France and is represented in brood stock used for stocking in the region. Hence, this strain has now introgressed into several French Mediterranean populations (Gil et al., 2016).

**Figure 2:**
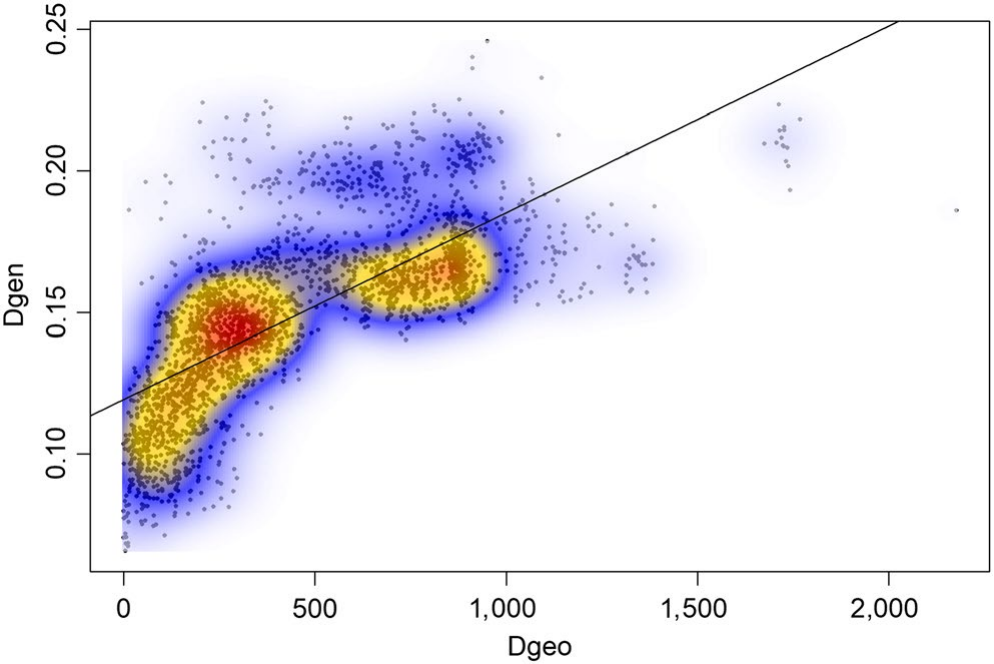
Relationship between geographical distance (Dgeo) between river mouths (in kilometres) and genetic differentiation (Dgen) estimated by Nei's distance, between pairwise collections of *Salmo trutta*. Colour contours indicate local kernel density estimates, where higher densities are shown by increasing degrees of red. The line indicating the least squares linear relationship between parameters is included for visual representation

Average Ɵ between temporal replicates within location was 0.011, ranging from 0.001 to 0.052 (Table S4). None exhibited statistically significant differentiation, except for the River Skals, where one replicate sample consisted of only 10 individuals, which may have biased allele frequency estimates. Forty‐three comparisons between spatial collections did not exhibit statistically significant differentiation (Table S4). In all cases, they represented collections from either neighbouring rivers or rivers from the same geographical area separated by less than 70 km. These included (a) Wadden Sea rivers Ribe, Kongeå and Sneum; Elbe and Weser, (b) Western Baltic rivers Krobæk and Mern, (c) British rivers Aln and Coquet, (d) Swedish Kattegat rivers Himleån and Fylleån, and (e) Swedish river Ätran and its tributary Högvadsån. Finally, Swedish Skagerrak collections spanning from River Hogarälv in north to Grannebyån in south generally exhibited lack of allele frequency differences (nos. 33–44 in Table 1). Pairwise differentiation between collections was positively associated with geographical distance (Monte Carlo randomization test; 999 randomizations: observed = 0.7157, *p* < .001, observed standard deviation = 12.868, expectation = −0.0002, variance = 0.0031). However, density analysis also suggested that IBD dynamics differed across geographical regions, as evidenced by the presence of at least two distinct clusters in the data (Figure 2). One of the clusters corresponded with comparisons between British and European/Scandinavian samples, as shown by the fact that one of two main clusters disappeared when British samples were excluded from analyses (Figure S2). When British samples were tested alone, two distinct clusters were suggested (Figure S3). Although sample size was somewhat limited encompassing 15 British collections in total, this suggested disjoint IBD dynamics with one strong north–south genetic break within this geographical region. A distinct separation between northern and southern British and all other populations was also evident in the PCA. Here, the three first principal components (PCs) described 5.3% of the variation, accounting for 2.4%, 1.6% and 1.3%, respectively (Figure S4). PC1 differentiated populations from continental Europe from all others, and PC2 separated SE British from NE British populations and Baltic Sea samples from other European samples, whereas PC3 mainly separated SE British samples from all others. In correspondence with a general IBD relationship, population sub‐structure was evident within each of the three main geographical regions, where individual genotype clustering to a large extent followed geographical relationships (Figures S5–S7). Genetic relationships within and between geographical regions are visualized in Figure 1a.

Using DAPC to determine population groupings returned most likely *k* = 10, with relatively similar BIC for *k* = 10–14, and genotype clustering strongly corresponding with geographical regions (Figure 1b). Expanding beyond 10 clusters identified increasing sub‐clustering within geographical areas, but with correspondingly decreasing posterior membership probabilities of individuals (not shown). DAPC returned overall good proportions of correct posterior assignment of individuals to the 10 clusters. Thus, between 0.50 and 1.00 (average = 0.91) of individuals per sample were assigned to a cluster including their collection origin. Hatchery admixed populations generally exhibited below‐average assignment to cluster origin (posterior assignment to cluster of origin in introgressed samples 0.50–0.91, average = 0.69), and when samples from introgressed populations were removed from analyses, average posterior assignment to cluster of origin increased to 0.95. A post hoc clustering analyses excluding all SNPs exhibiting positive outlier behaviour (see below) returned the same number of, and individual genotype affiliations with, population clusters as did analysis including all SNPs (not shown).

### 3.3 Identification of SNP outliers

**Figure 3:**
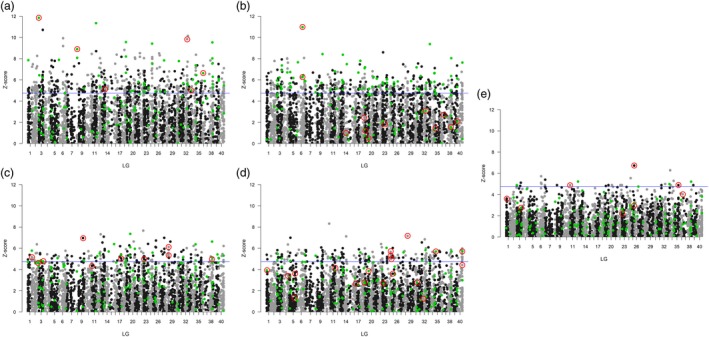
Manhattan plot showing *z*‐values from gene–environment association (GEA) analyses for 3,629 SNP loci aligned by position in LG 1–40. Individual plots a–e show *z*‐values associated with each SNP for each of the five composite environmental variables Dim1‐5. SNPs above the blue horizontal line show statistically significant GEA. Green symbols indicate 138 positive outlier SNPs identified in both population sub‐sets with *pcadapt* and/or *bayescan*. SNP loci showing association with individual variables in RDA are circled in red

The *pcadapt* analyses comprising all samples showed agreement with the results using *adegenet* by providing the optimal model resolution when grouping genotypes into 10 clusters (Figure S8). When controlling for population clustering, *pcadapt* identified 24 outlier SNPs distributed across 13 LGs, which was considerable lower than the global result from *bayescan*, which identified 576 outlier SNPs (183 with lower and 393 with higher than expected divergence, Table S3). Seventeen outlier SNPs were identified with both methods. Comparing *pcadapt* outliers detected in paired sub‐sets of data, 11, 11 and 17 outliers were identified in sub‐set 1, sub‐set 2 and sub‐set 1 and 2 combined, respectively. There was good correspondence in the numbers of geographical clusters identified for the three data sets (Figure S9), but relatively little overlap between loci identified as outliers in the sub‐sets 1 and 2 (four of 11 outlier loci, Table S3, Figure S10). The low overlap in outliers detected with different sub‐sets of samples was also evident with the *bayescan* approach, where just 137 of 401 outlier loci (34%) were identified in both sub‐sets 1 and 2 (Table S3, Figure S11). Outlier SNPs identified with *bayescan* were distributed across all LGs (Figure 3, Table S3). There was no trend for covariance between LG size and numbers of outliers identified and no apparent clustering of outliers within LG.

### 3.4 Genotype associations with environment

**Figure 4:**
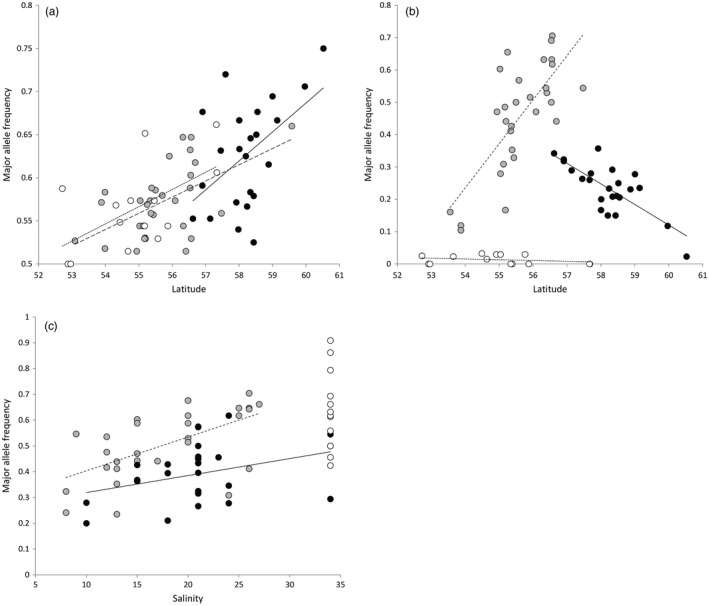
Association between environmental variables and frequency of major allele for populations in the three geographical regions Britain (open symbols, dotted line), Scandinavian peninsula (black symbols, solid line) and Continental Europe (grey symbols, stippled line), exemplifying gene–environment association relationships that are as follows: (a) specific to geographical regions; locus “Gdist_S82886_3406,” identified as selective outlier in both *pcadapt* and *bayescan*, (b) general across regions; locus “cDNA_S415600_5145,” showing no outlier behaviour but association with temperature parameters and latitude (shown here) or (c) general across regions characterized by salinity gradients, but not in the salinity‐invariant region Britain; locus “cDNA_S84920_5746.” None of the three loci exhibited statistically significant outlier behaviour in RDA (see text). Least squares regression lines are shown to guide inference

Principal component analysis of spatial and environmental variables showed that the five top PCs explained 69% of the variation (Figure S12). PC1 (32% variation explained) described parameters distinguishing the three main geographical regions, Britain, Continental Europe and Scandinavian Peninsula, including temperature (higher for Britain) and salinity (lower for Continental European samples). PC2 (15%) was associated primarily with climatic variables related to precipitation. PC3 (10%) was associated with soil pH. PC4 (6%) was associated with high summer temperatures, and PC5 (5%) was associated with soil type (Tables S5 and S6). Varying the number of latent factors in the GEA model affects identification of loci associated with parameters (Frichot et al., 2013), which may particularly influence inference if population structure follows an IBD model and number of population clusters is ambiguous. In our analysis, 997 loci showed GEA when *K* was set to 14 population clusters, all of which were a sub‐set of loci identified at *K* = 9–13. We therefore report GEA results for *K* = 14 to reduce rates of false positives. The majority of loci (89%) showed association (at *z* > 4.7) with a single variable. There was generally low overlap between loci showing GEA with the tested variables and loci identified as outliers, and just 13 loci were identified in all three tests. All LGs contained GEA loci (ranging 4–50 loci, average 25, per LG; Figure 3). The majority of GEA were observed with variables differing among the three geographical regions (421 PC1 associations) and with temperature/soil variables (371 PC2 associations). GEA was less often related to geochemistry (207 PC3 associations), maximum temperature/precipitation (172 PC4 associations) and soil type (33 PC5 associations). There were several instances of interaction between GEA and geographical region. Thus, for 11 loci coming out as statistical outliers in *pcadapt* and showing GEA, there was a strong effect of geographical region on relationships between allele frequencies and environmental variables. This suggests that associations are lineage specific and potentially confounded by the phylogenetic history of populations. An example of a typical region‐specific GEA relationship is shown in Figure 4a. In other cases, GEA was observed across all three main geographical regions, suggesting that loci were affected in the same direction by specific selective pressures across demographic lineages (exemplified in Figure 4b). The locus showing the strongest association with salinity exhibited concurrent relationships in the two geographical regions bordering the North Sea–Baltic Sea transition zone describing a salinity gradient varying from fully saline waters (34 ppt) to brackish conditions (8 ppt), but also large allele frequency variation within the more salinity‐invariant British populations (Figure 4c).

**Figure 5:**
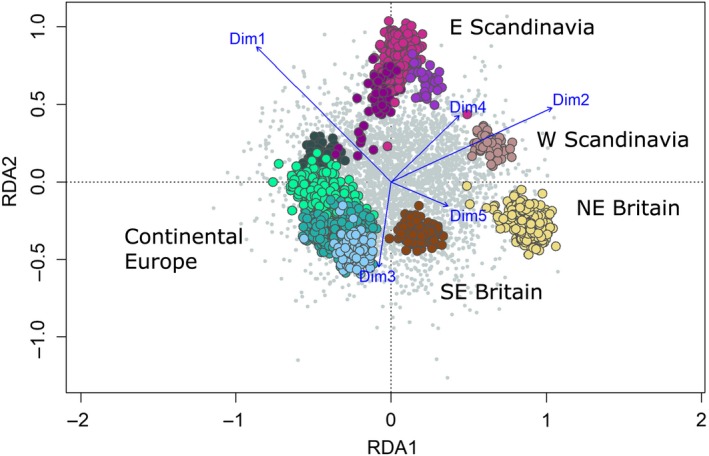
Redundancy analysis results showing the first two axes, explaining, respectively, 40% and 27% variation. Grey points indicate individual SNPs, and dots indicate individual fish colour coded by their geographical region following notation in Figure 1 (Continental Europe: blues and greens; E Scandinavian Peninsula: purples; W Scandinavian Peninsula: pink; NE Britain: light yellow; SE Britain: brown). Blue vectors represent the environmental predictors represented by the five composite environmental PCs. Both SNP and individual fish scores are scaled symmetrically by the square root of the eigenvalues

In the RDA, the first three components explained, respectively, 40%, 27% and 18% of the variation, and the five composite environmental parameters all showed statistically significant variation with genotypes (all *p* < .001). The first three axes separated, respectively, (a) British and West Norwegian populations, (b) Scandinavian populations and (c) SE British populations. As in LFMM analyses, PC1 (mainly associated with geography and temperature variables) showed the strongest association, followed by PC2, associated with precipitation and soil type (Figure 5). When testing which SNPs were most strongly associated with each of the five parameters, respectively, 6, 12, 10, 19 and 8 SNPs (55 SNPs in total) came out with high loading values for PC1–5. Correlations between these SNPs and their most strongly associated environmental parameters were not marked (average *r* = .29, range .15–.49). Thirty‐nine of the 55 SNPs (71%) identified as showing GEA in RDA also showed GEA in LFMM (Table S3).

## 4 DISCUSSION

Our study has two main merits. Firstly, by taking advantage of genomic resources developed for *S. trutta*, this study provides the most detailed examination of large‐scale genetic structure in a central part of the species' native range. We identified very strong sample clustering corresponding with broad geographical regions, as well as clear genetic breaks among samples within regions, that in several cases were associated with climatic variables. Secondly, we contribute to the evaluation of state‐of‐the‐art statistical approaches for identifying genetic signatures of selection and their association with environments, through reporting on genome‐wide detection of loci exhibiting GEA and showing that discovery in some cases was strongly dependent on statistical method and sampling design.

### 4.1 Inference from genome‐wide SNP variation in *S. trutta*

Genomic resources are rapidly growing for *S. trutta* (Carruthers et al., 2018; Leitwein et al., 2017; Lemopoulos et al., 2018), but our study is the first to apply genome‐wide SNP data to examine broadscale population relationships and associations with evolutionary drivers across regional scales. The stringent criteria for selecting SNPs are likely to have caused some ascertainment bias, thereby skewing MAF towards higher values and lowering power to disentangle effects of neutral versus selective processes. Ascertainment bias in SNP selection may bias inferences of population demography (Guillot & Foll, 2009, but see also Albrechtsen, Nielsen, & Nielsen, 2010). In this study, individuals from all major genetic clusters detected were represented in the ascertainment panel, suggesting that our assessment of spatial structure is unlikely to suffer substantial bias with respect to the magnitude of genetic differences among populations, at least across larger geographical scales. Individuals in the SNP development ascertainment panel represented both the NE British and the SE British population clusters. The lower overall genetic diversity observed in samples from Britain was thus unlikely an artefact of ascertainment bias. Although methods have been proposed to correct for ascertainment bias (Guillot & Foll, 2009), most require some assumption about allele frequency distributions under un‐biased conditions. Lacking such information, we therefore refrained from estimating demographic parameters, such as phylogenetic relationships and divergence times. Yet, we emphasize that given the genomic coverage and quantity of markers, the relative magnitudes of differentiation detected among samples are expected to be robust.

### 4.2 Geographical breaks in population structure

Our results support previous inference based on smaller marker panels and samples with more restricted geographical coverage. Accordingly, this study reinforces the notion that trout commonly show temporally stable (at least on a decadal scale) genetic structure on local river or neighbouring watershed scales and that the distribution of genetic variation can often be described by isolation‐by‐distance dynamics, attributed to the species' homing instinct (Griffiths, Koizumi, Bright, & Stevens, 2009; Meier et al., 2011; Stelkens, Jaffuel, Escher, & Wedekind, 2012). Although our sampling scheme was not exhaustive, we benefitted from substantial geographical sample coverage allowing fine‐scale inference about structural relationships from wide to small geographical scales. First, we identified strong genetic breaks among populations inhabiting the landmasses Britain, the European continent and the Scandinavian Peninsula, most likely reflecting allopatric divergence during postglacial colonization events (Bernatchez, 2001; Cortey, Vera, Pla, & García‐marín, 2009; Hewitt, 2000).

A novel result was the identification of strong divergence between northern and southern British populations. Our sampling design did not allow for precise definition of the geographical break between the two clusters of samples, nor whether geographically intermediate populations constituted a hybrid zone between clusters. Analyses of mitochondrial DNA markers have not identified genetic breaks between populations sampled in south and north (McKeown, Hynes, Duguid, Ferguson, & Prodohl, 2010). However, complex phylogeographical processes and incomplete lineage sorting could have affected this result (McKeown et al., 2010) masking the divergence observed with nuclear markers. In contrast to Northern Britain, the area spanning from Wales in west to east Anglia in east was ice‐free during the last glaciation and thus has a different geological history from that of the north. Moreover, the entire Southeast Britain previously constituted the catchment area for the River Thames draining into the southern North Sea (Rose, 1994). Analyses of additional samples from the River Thames and from rivers draining into the English Channel support the existence of close genetic relationships among SE British trout populations (D. Bekkevold, A. King, J. Stevens, unpublished results). Trout populations in SE Britain are generally characterized by inhabiting short, lowland rivers, whereas NE British populations included in our study generally inhabit larger upland rivers with stronger gradients and higher water flow. The genetic discontinuity identified in our study is hence in agreement with expectations for two population clusters inhabiting different environments and having discrete evolutionary histories potentially associated with selective sweeps that maintain separate demographics to present day.

We identified another prominent genetic break between populations from the Scandinavian Peninsula and Continental Europe (including the Danish Belt Sea islands). There was apparently a strong barrier to genetic exchange between rivers draining into east and west of the Skagerrak and Kattegat seas, in some places separated by less than 60‐km waterway. Such strong genetic separation is remarkable, given the species' propensity for long distance feeding migrations (Koljonen, Gross, & Koskiniemi, 2014), including within the Kattegat area (D. Bekkevold, unpublished data), and points to accurate homing to specific geographical regions.

As expected, genetic structure was also evident within regional population clusters. In most cases, genome‐wide differentiation followed geographies in correspondence with founder events combined with isolation‐by‐distance dynamics. Nonetheless, we stress that although inference about overall genetic relationships among populations should be robust, the potential ascertainment bias of the applied markers could somewhat influence the inference of the underlying demographic processes. Even so, the markers analysed here will be useful for delineating conservation units and their distributions in time and space, as well as for individual assignment and identification of population admixture (Nielsen et al., 2012).

### 4.3 Contrasting results from different genome scan approaches

We used one of the, to our knowledge, largest sample sizes to date for outlier analysis across a large number of geographically widespread populations and typed more than 3,000 SNPs, which is the suggested threshold to limit false discovery bias (Ahrens et al., 2018). The application of two different genome scan methodologies allowed further insights into the processes hypothesized to drive population differentiation. The overarching result from both methodologies was the finding of statistical outliers across broad expanses of the trout genome. Large blocks of highly divergent loci positioned in one or a few linkage groups (sometimes referred to as “genomic islands” often attributed to chromosomal structures such as inversions) have been identified to differentiate ecotypes in fish (Bradbury et al., 2013; Hemmer‐Hansen et al., 2013; Lamichhaney et al., 2017). However, unless a population has undergone a recent selective sweep, local selection is unlikely to act on only a few large‐effect loci or genomic regions (Rockman, 2012). Although the genetic structures we observe likely also reflect drift and, potentially, postglacial secondary contact between lineages, our results are thus in line with the pattern from other salmonids (Bourret et al., 2013; Pritchard et al., 2018) that populations exhibit genomic variation indicative of multiple sweeps and divergent selection acting on broad expanses of the genome.

When all collections were included in global analyses, the two methodologies returned large numerical differences in detected outlier loci. Thus, *bayescan* identified 11% outliers, whereas when controlling for regional population clustering, *pcadapt* identified less than one per cent outliers. Albeit both figures at face value differ from estimates of ~5% outlier loci seen in other species, they are still within the reported range (reviewed in Ahrens et al., 2018). Our analysis is also consistent with results being dependent on both statistical methods used (Bradbury et al., 2013) and the type of genetic variation studied (de Villemereuil et al., 2014; Vasemägi, Nilsson, & Primmer, 2005; but see Ahrens et al., 2018). The *bayescan* approach may yield low detection power under an “isolation with migration” model, as is expected for brown trout populations, and sampling large numbers of genotypes may concurrently inflate numbers of false positives (de Villemereuil et al., 2014). The population clustering parameter (*k*) applied in our *pcadapt* analysis was based on all loci, rather than restricted to sub‐sets of loci presumed to reflect neutral demographic processes. This could potentially have reduced number of outliers detected with the *pcadapt* approach, compared to the “demography‐naïve” *bayescan* approach. Conversely, in a minor number of cases, paired rivers did not exhibit statistically significant divergence, which could have led to slightly increased numbers of false positives identified across sub‐set analyses. All of these error sources may have contributed to the observed discrepancy between the two outlier analysis approaches. The inference gained in our study from comparing methods is that outliers may be highly specific to hierarchical population clusters, as evidenced by the much lower number loci detected with the *pcadapt* approach incorporating demography than in the approach not incorporating demography. The relatively low overlap in outliers identified in sample sub‐sets corroborates the view that sampling design may have a strong effect on detection of genomic regions underlying selection. However, when comparing outliers identified across both sample sub‐sets, all four *pcadapt* outliers and 90% of 137 *bayescan* outliers also showed GEA. This supports the notions that comparing results from different statistical methods (de Villemereuil et al., 2014) and applying a paired‐population sampling design (Lotterhos & Whitlock, 2015) can strengthen inference about selective processes under a hierarchical population scenario.

### 4.4 Genotype‐environment associations within and among clusters

We identified several GEA related to climatic variables temperature and precipitation, which is consistent with results in other fish species, including other salmonids (Bourret et al., 2013; Chen, Farrell, Matala, & Narum, 2018; Hecht, Matala, Hess, & Narum, 2015; Matala, Ackerman, Campbell, & Narum, 2014; Perrier, Bourret, Kent, & Bernatchez, 2013). Such general relationships are not surprising given that in fishes, temperature is linked to key physiological, developmental and behavioural processes, rendering fish highly sensitive to climatic and thermal conditions (Crozier & Hutchings, 2014; Eliason et al., 2011). Climatic drivers are hence expected to exert selection pressures on local populations, also in trout (Jensen et al., 2008), although some salmonid studies indicate stronger effect of phenotypic plasticity rather than adaptation to specific temperatures (Solberg, Dyrhovden, Matre, & Glover, 2016). Nonetheless, temperature and precipitation are likely to be correlated with other, untested, environmental variables, and short of experimental manipulations, GEA studies can only be indicative of drivers underlying local adaptation (McCairns, Smith, Sasaki, Bernatchez, & Beheregaray, 2016). LD, especially high within salmonids, is likely to show low decay across broad genomic regions, also obscuring the direct relationship between specific SNP's and environmental variables. Thus, confirmation of functional and adaptive significance of post hoc identified genes requires rigorous testing, and although several GEA studies report annotation of SNPs found to be associated with environmental variables, we follow the argumentation in Pavlidis, Jensen, Stephan, and Stamatakis (2012) and refrain from mining for annotation of GEA markers. In GEA testing, both the univariate latent factor mixed model approach LFMM and the ordination‐based method RDA identified loci associated with environmental parameters, but the latter method identified less than 5% of the numbers of SNPs as the former. This differs from results of a recent study in *Populus*, where RDA had superior statistical power and showed lower nondetection rates than LFMM (Capblancq et al., 2018). That study also found that the two approaches did not consistently identify known QTLs, whereas in our study, SNPs showing GEA with RDA generally also were identified with LFMM. Interestingly, there was relatively stronger consistency in loci identified with both methods to be associated with the variable Dim1 that comprised the main differences among geographical regions than across analyses for Dim2‐5, associated with environmental variables that were less specific to individual geographical regions. This indicates that GEA methods were most consistent for variables showing the strongest inter‐regional divergence. Numbers of GEA identified with LFMM were high (totalling almost 30% of all loci) and spread across all LGs. Although results were controlled for inflation of false positives, this suggests that absolute numbers of GEA identified may have been upwardly biased. Irrespective of a potential bias, the identification GEA across broad expanses of the trout genome with both methods is suggestive of locally adapted variation being pervasive throughout multiple genomic regions. Our results may also reflect the expectation that locally adapted traits often are polygenic and governed by loci that individually exhibit low effect that are difficult to detect statistically (Savolainen et al., 2015).

A strength of our sampling design is that it represents a paired‐gradient design, in the sense that it allowed for an assessment of whether the same loci were associated with climatic gradients across presumably allopatric population clusters. Although several loci showed consistent relationships with environmental variables, there were also several cases where relationships were evident within only one or two population clusters. This was exemplified by the locus showing maximal association with salinity. Here, both the Scandinavian and the European mainland population clusters, which co‐habit the North Sea–Baltic Sea salinity gradient, were found to display increasing allele frequencies with decreasing salinity, although variance among populations was pronounced. For the same locus, allele frequencies also varied greatly across the British populations that invariantly inhabit rivers, which drain into high‐salinity coastal environments. This result could indicate that salinity conditions, rather than demography alone, drive dynamics in that locus in Scandinavian and European mainland populations, and that neutral, or at least dissimilar, dynamics drive allele frequencies in British populations. The application of composite environmental variables in the GEA models, rather than examining single, in several cases inter‐correlated variables individually may have obscured identification of strong relationships between specific variables and SNPs. However, in the present context association between environments and genomic regions suggests that adaptation to local environments may be complex but is pervasive across populations.

### 4.5 Genetic management of brown trout

Although genomic analysis and identification of adaptive variation is not a requirement for conservation per se (Flanagan, Forester, Latch, Aitken, & Hoban, 2018), our results have direct management implications. First, our results can be applied to define conservation units and to prioritize management actions (Funk, McKay, Hohenlohe, & Allendorf, 2012; also see discussion in Mee, Bernatchez, Reist, Rogers, & Taylor, 2015). Specifically in salmonids, supplementary stocking has been a popular management tool to mitigate dwindling populations in the face of habitat deterioration and fisheries exploitation. A substantial body of literature has addressed potential genetic effects of stocking non‐native genetic material into wild populations (Laikre, Schwartz, Waples, & Ryman, 2010) where effects on population fitness are mainly expected to be negative (Edmands, 2007). Our SNP data represent a valuable genomic resource for assessment and monitoring of introgression in local populations (Glover et al., 2013; Vera et al., 2018) and as a tool to design experimental tests of fitness effects of introgression (Hagen et al., 2019). A practical advantage of our SNP array approach, in comparison with, for example RAD and other genotyping‐by‐sequencing‐based approaches (Andrews, Good, Miller, Luikart, & Hohenlohe, 2016), is that our markers are directly transferrable between genotyping platforms and that information can be used to tailor analyses addressing specific management objectives. To maximize the efficiency of conservation efforts, there is increasing effort to tailor releases by using the genetically most suitable stocking material (Caudron, Champigneulle, Guyomard, & Largiader, 2011). Genomic coverage was relatively low in our study, and the knowledge gained here is unlikely to fully reflect functionally significant differentiation within and among populations. It is therefore possible that some of the populations exhibiting no genetic differentiation in fact are locally adapted. Moreover, our sampling design was not equally comprehensive across all geographical regions. Although our study thus has shortcomings, it nonetheless provides essential information on the geographical distribution of populations more likely to share evolutionary histories that would allow for successful reintroductions, where needed. We identified geographical regions exhibiting overall weak genetic differentiation among neighbouring rivers, as, for example, was the case for several Swedish Skagerrak rivers. Where human‐mediated gene flow can be discounted, genetically similar populations are inferred to also display stronger demographic connectivity. Results could indicate that population dynamics can be described in a meta‐population context, where demographic stability may be dependent on regional, rather than local processes (Østergaard, Hansen, Loeschcke, & Nielsen, 2003). Especially in systems consisting of small, temporally unpredictable streams (in terms of discharge), straying between neighbouring rivers might be a strategy that has been favoured by natural selection. Conversely, in a number of cases, river populations within a relatively constrained geographical area exhibited marked genetic divergence, suggestive of selection against interbreeding. In such cases, restocking activities should refrain from mixing gene pools, which could result in introgression and outbreeding depression. Although extensive genomic and experimental analyses are required to predict the suitability of directed releases of specific non‐native strains, most applied conservation work relies on the establishment of practical guidelines that do not require detailed case‐by‐case study. An applied conservation guideline adhered to in Denmark is that restocking material should represent fish of the genetically closest related population and from the geographically closest population if that information is not available (Berg & Hansen, 1998). Governance of salmonid stocking varies strongly among north‐east Atlantic legislative units, and there is a call for increased attention to halt unsustainable management practises (Aas et al., 2018). Our results serve as a tool that can be directly implemented in outlining conservation units and to advice on the geographical distribution of genetic populations that can be expected to be suitable for preserving adaptive state, for example under local restocking activities.

## CONFLICT OF INTEREST

None declared.

## Data Availability

SNP genotype data are deposited at https://data.dtu.dk/10.11583/DTU.10008020.
