# Supplementary material for: Northern European Salmo trutta (L.) populations are genetically divergent across geographical regions and environmental gradients
Source: Evol Appl. 2019 Nov 1;13(2):400–16. doi: 10.1111/eva.12877 (PMC6976966; doi:10.1111/eva.12877)
Supplement: Supplementary file 1 [file EVA-13-400-s001.docx]

**Supplementary Figures for manuscript EVA-2019-107-OA:**


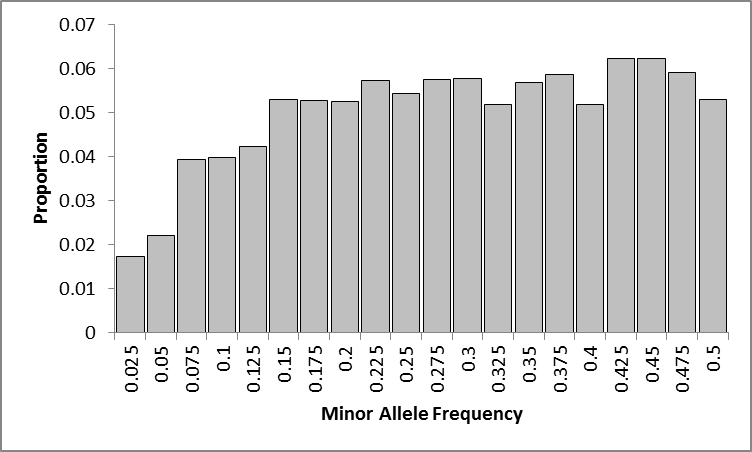


Supplementary Figure S1. Distribution of MAF in 3782 SNP loci typed in 2510 trout from 83 collections.


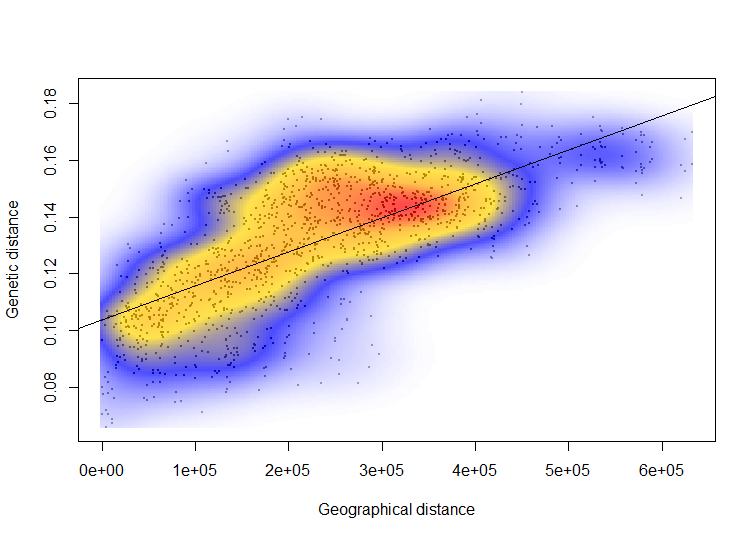


Supplementary Figure S2. Relationship between genetic and geographic distances (in meters) in Continental Europe and Scandinavian Peninsula collections (N=54), i.e. excluding collections from Britain. Monte-Carlo randomisation test (999 randomisations): Obs = 0.7005, P<0.001, standard deviation OBS = 13.458, expectation 0.000502, variance = 0.002705.


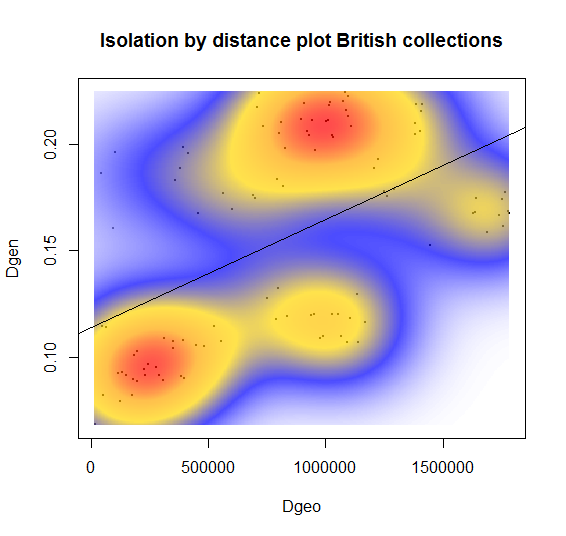


Supplementary Figure S3. Relationship between genetic and geographic distances (in m) in British collections (N=15). Monte-Carlo randomisation test (999 randomisations): Obs = 0.5196, P=0.007, standard deviation OBS = 2.8560, expectation -0.00154, variance = 0.033289.


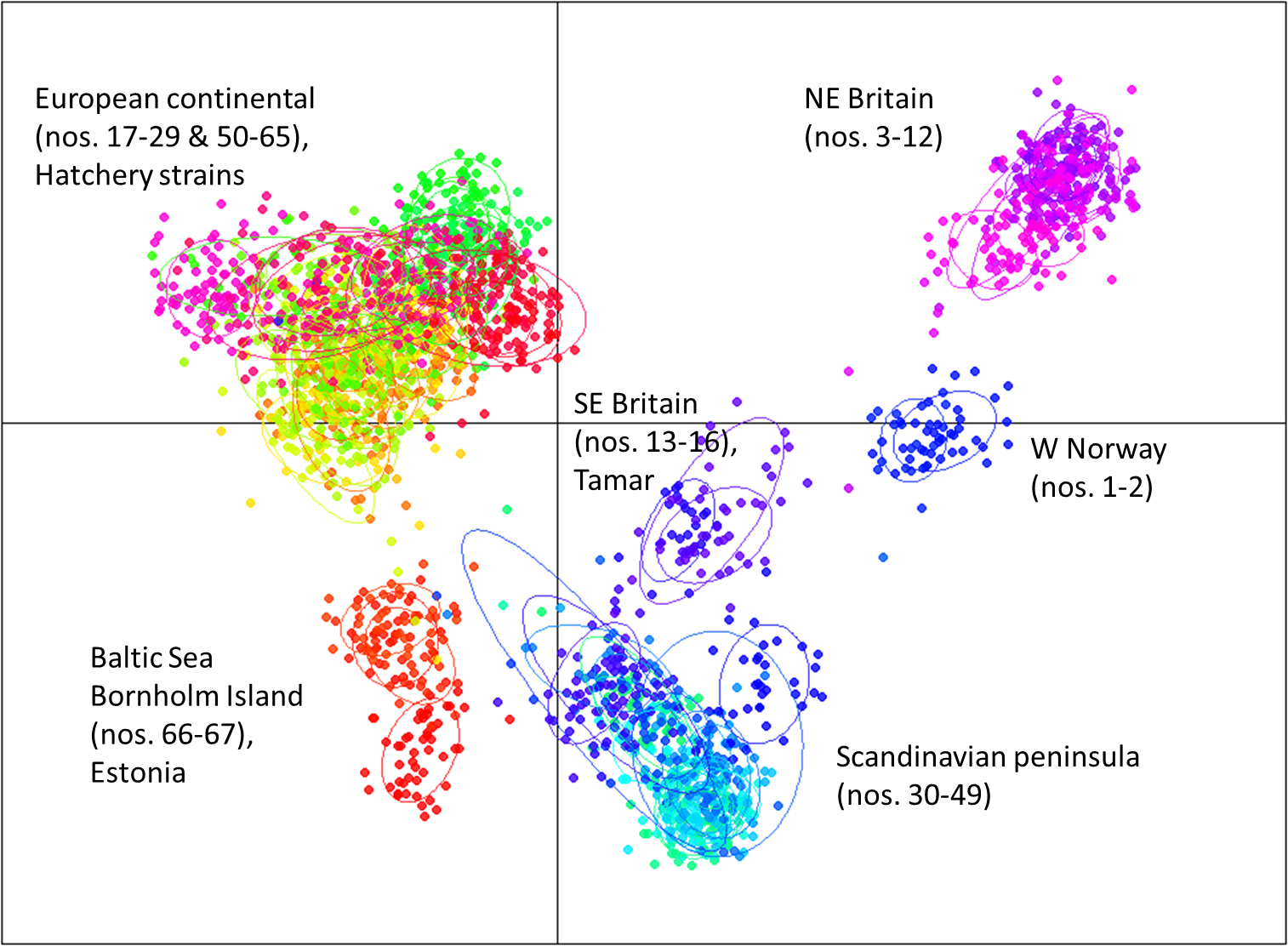

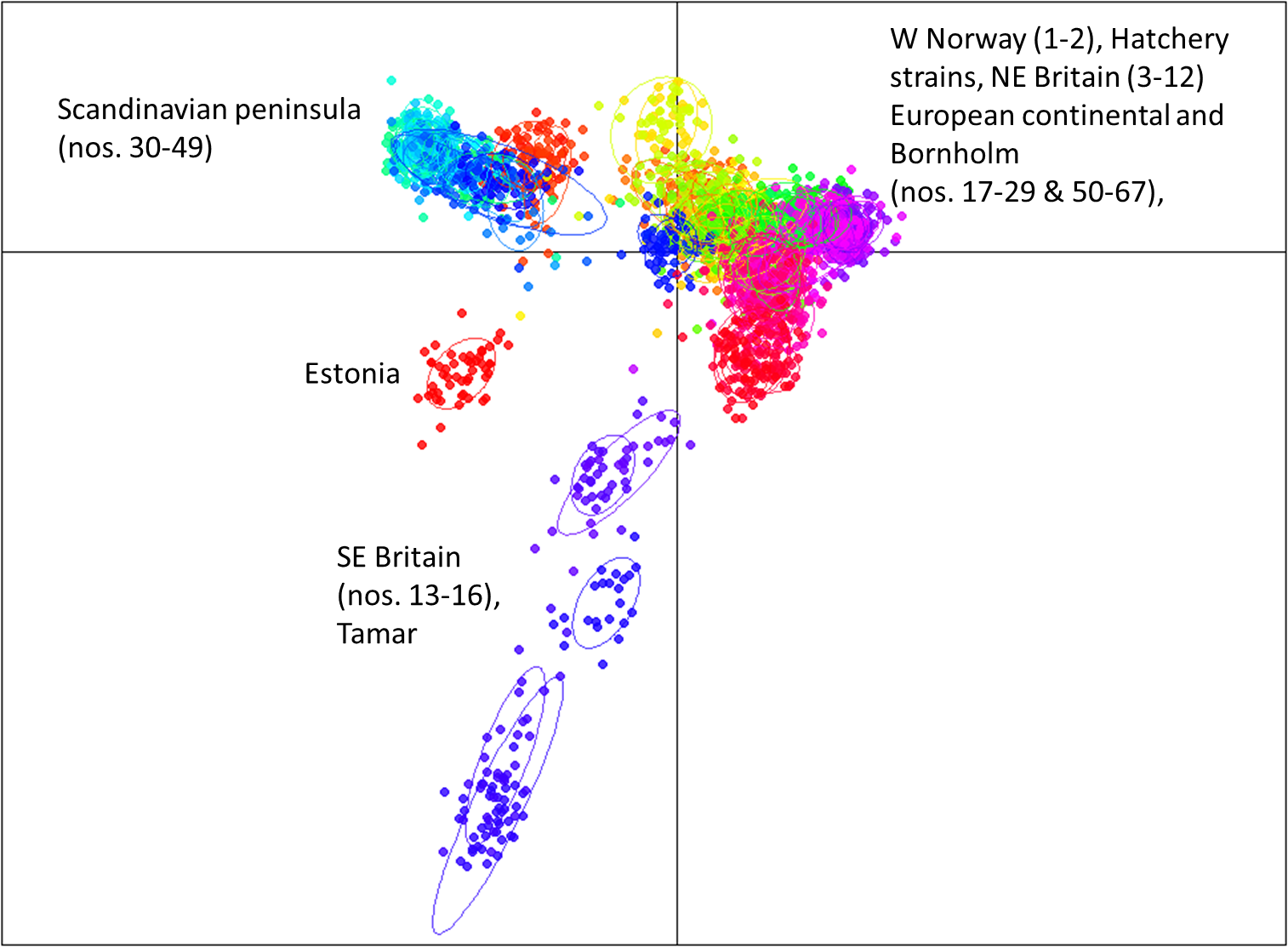


Supplementary Figure S4. PCA plot 68 brown trout samples and 3782 SNPs showing a) PC1 and PC2, explaining respectively 2.37% and 1.61% variation, and 2) PC2 and PC3 (explaining 1.3% variation). Individual samples are indicated by different colour symbols and main population clusters are indicated by their geographical relationships.


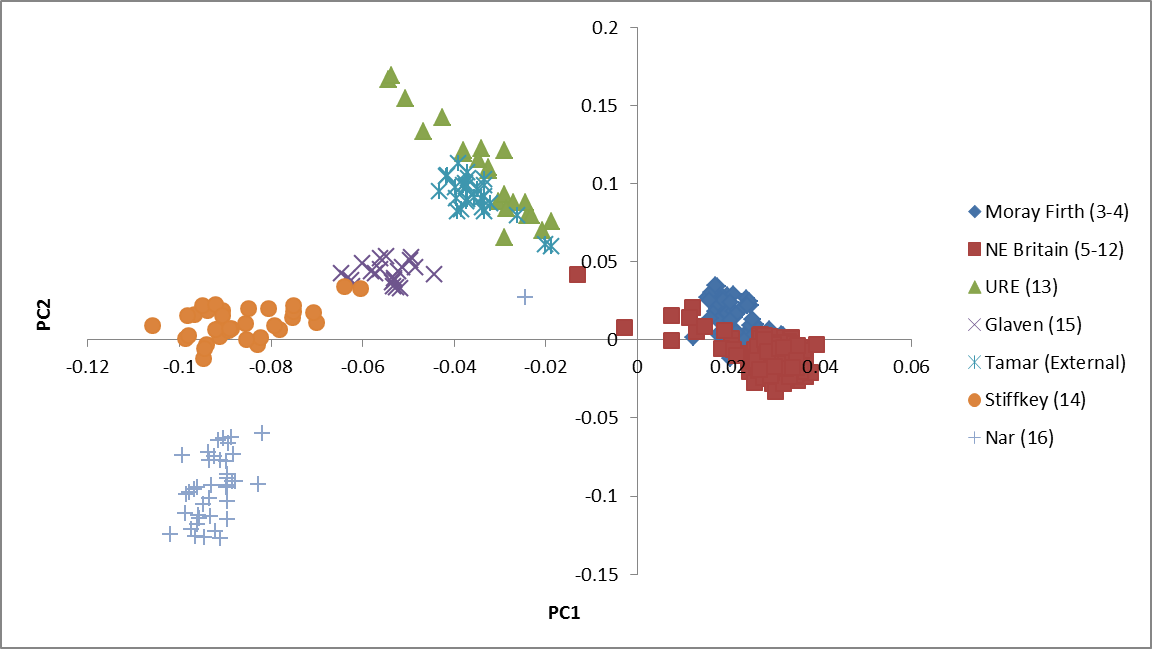


Supplementary figure S5. PCA results reflecting sub-clustering of individual fish collected in Britain. PC1 tracks collections from North versus South of latitude 54°N, and PC2 mainly differentiates among collections from SE Britain. Numbers in brackets refer to collections in Table 1 and symbols identify collections with similar clustering behavior.


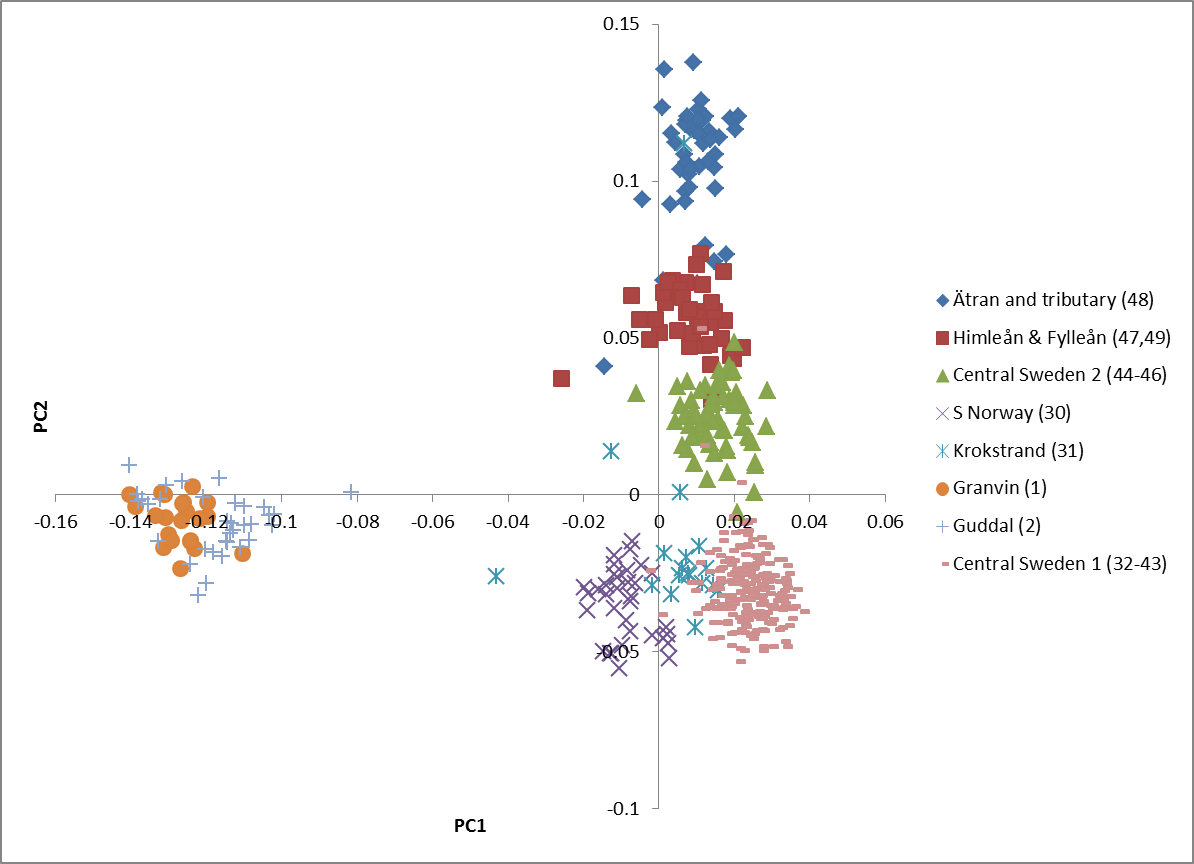


Supplementary Figure S6. PCA results reflecting sub-clustering of individual fish collected in the Scandinavian peninsula. PC1 mainly tracks collections from West to East, and PC2 mainly track collections from South to North. Numbers refer to collections in Table 1 and symbols identify collections with similar clustering behavior.


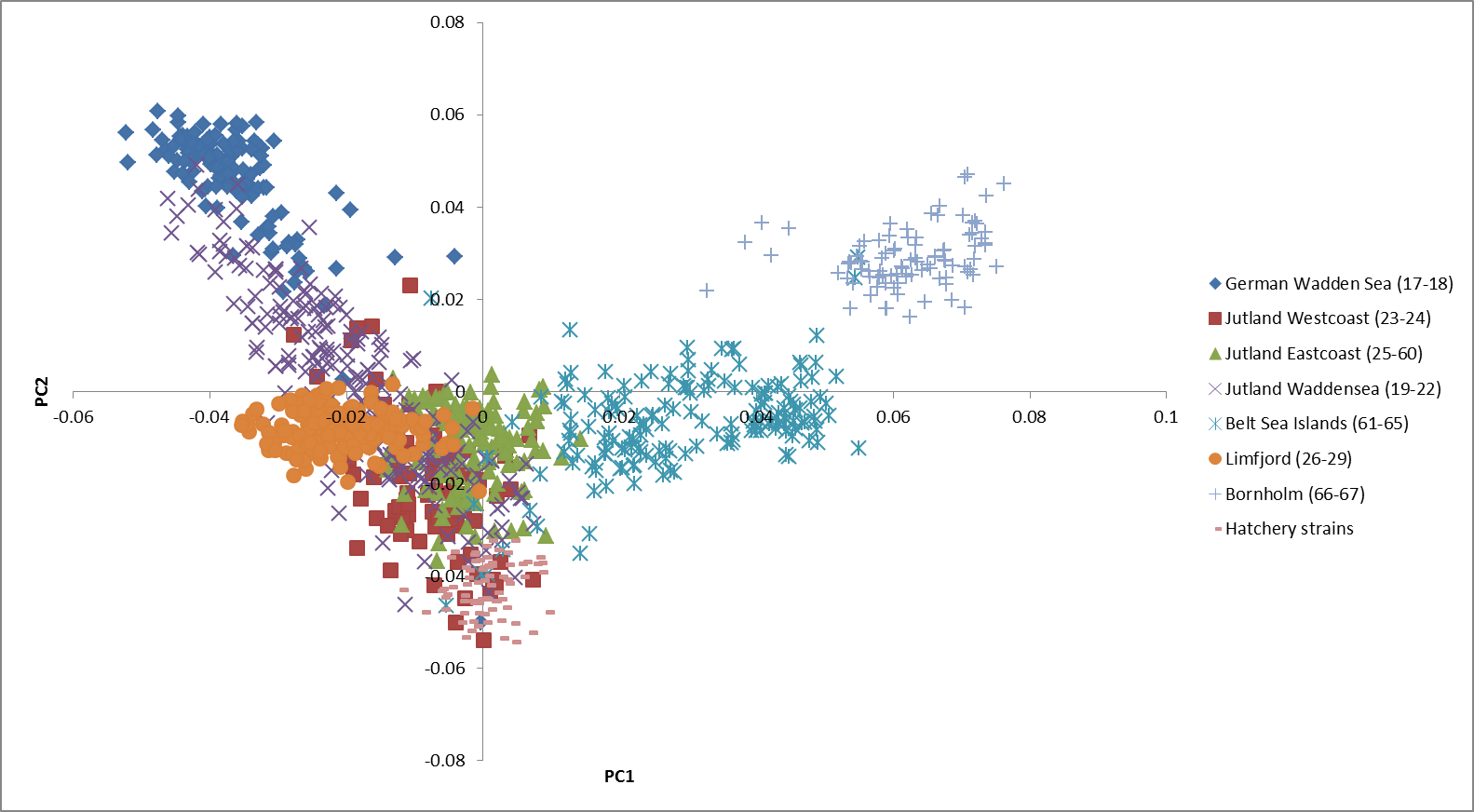


Supplementary Figure S7. PCA results reflecting sub-clustering of individual fish collected from Continental Europe (and Danish Belt Sea islands). PC1 tracks collections from West to East, and PC2 mainly reflects collections from South to North. Numbers in brackets refer to specific collections and symbols identify geographical regions of collections (see Table 1). Samples include temporal replicates as well as introgressed populations.


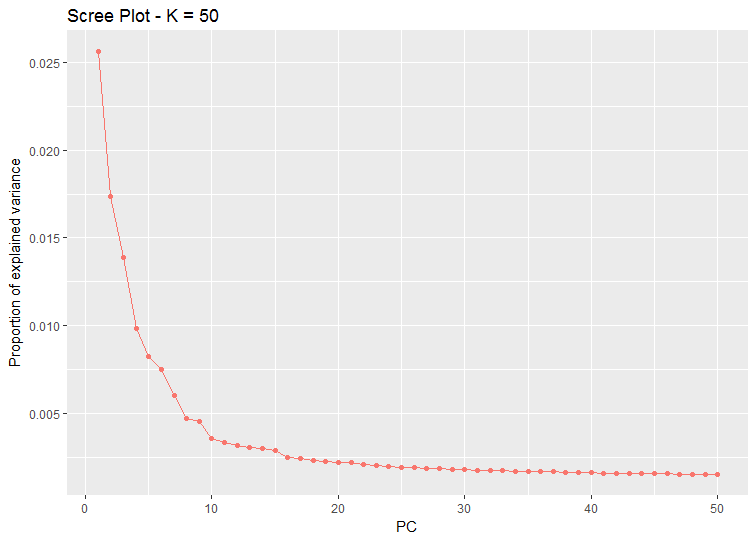


Supplementary Figure S8. Scree plot from *pcadapt* PCA with 3782 SNPs and 67 collections (i.e. excluding external collections from France, Cornwall and Estonia as well as temporal replicates).


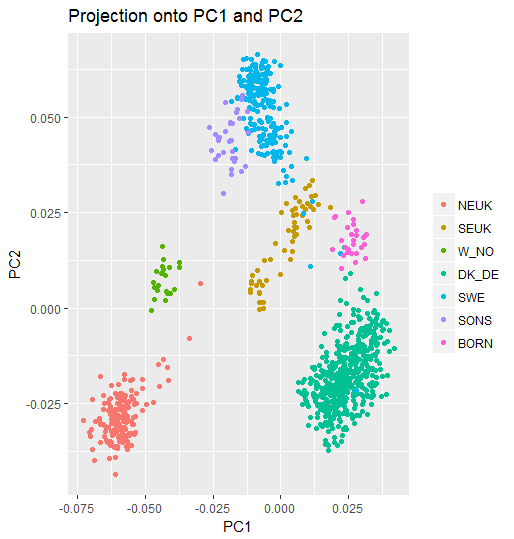

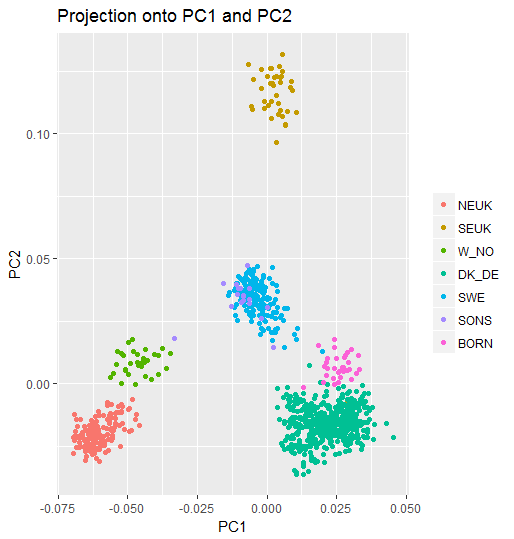


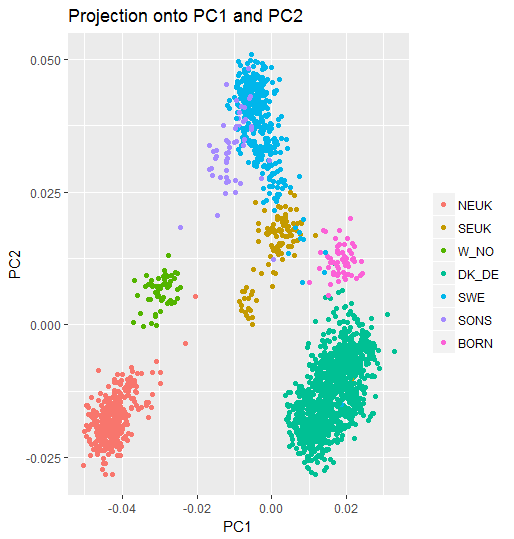


Supplementary Figure S9. PCA plot showing first two PCs for (k=9) for, respectively sample sub-sets, 1, 2 and 1 and 2 combined. Individuals’ geographic region of origin is indicated by symbol colouring as follows: NE Britain (salmon pink), SE Britain (light brown), W Scandinavian Peninsula (warm green), E Scandinavian Peninsula (light blue and dark purple), Continental Europe (cold green), and W Baltic Bornholm island (pink).


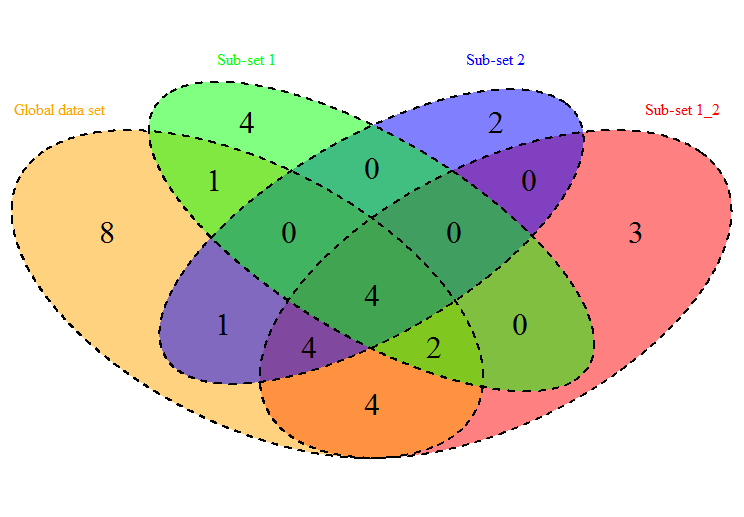


Supplementary Figure S10. Venn diagram showing overlap in loci detected as positive outliers in *pcadapt* analyses for data sets with different numbers of samples, where ‘Global data set’ includes all 74 spatial collections, ‘Sub-set 1’ includes 34 collections, ‘Sub-set 2’ includes the other 34 half of the sample pairs, and ‘Sub-set 1_2’ includes both latter sets (N=68). See Table 1 for specific spatial collections going into analyses.


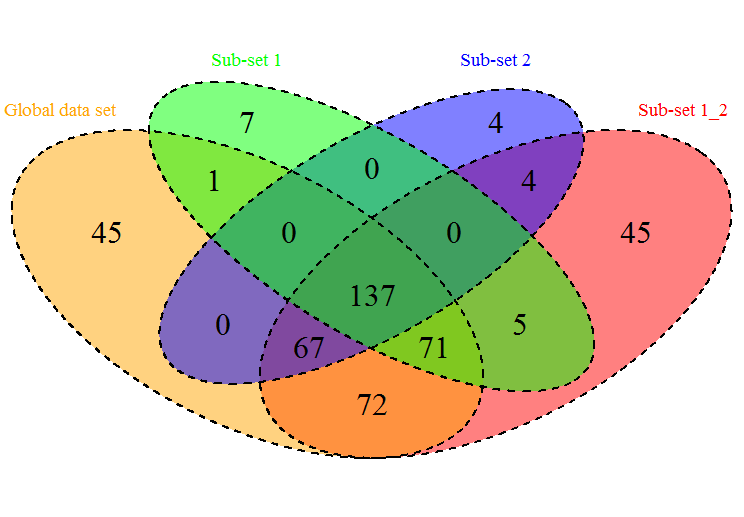


Supplementary Figure S11. Venn diagram showing overlap in loci detected as positive outliers in *bayescan* analyses for data sets with different numbers of collections, where ‘Global data set’ includes all 74 spatial collections, ‘Sub-set 1’ includes 34 collections, ‘Sub-set 2’ includes the other 34 half of the collection pairs, and ‘Sub-set 1_2’ includes both latter sets (N=68). See Table 1 for specific collections going into analyses.


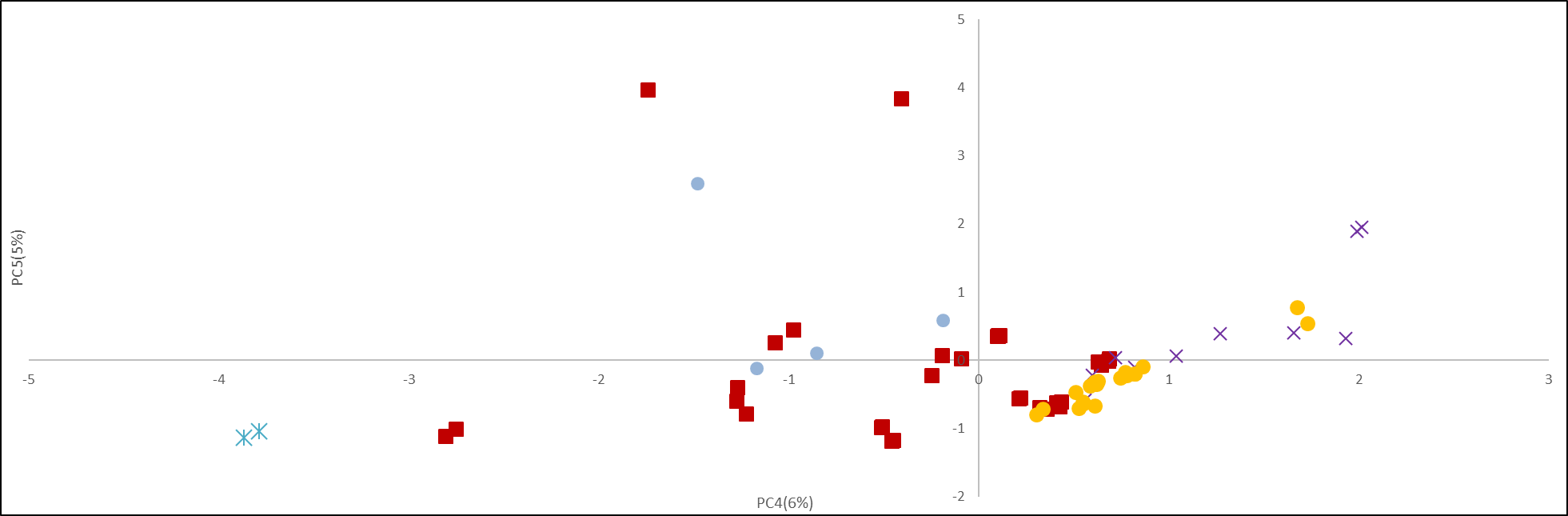


Supplementary Figure S12. PCA plots showing clustering of population samples (main geographical regions in different symbols: red squares: Continental Europe, blue circles: SE Britain, orange circles: Eastern Scandinavian peninsula; purple crosses: NE Britain, blue stars: Western Scandinavian peninsula) by 15 environmental variables for PC1-5. The proportion variance explained by PC1-5 is shown in brackets next to axes.
